# A critical review of rheological models in self-compacting concrete for sustainable structures

**DOI:** 10.1038/s41598-023-48673-6

**Published:** 2023-12-02

**Authors:** Kennedy C. Onyelowe, Denise-Penelope N. Kontoni

**Affiliations:** 1 Department of Civil Engineering, School of Engineering, University of the Peloponnese, 26334 Patras, Greece; 2 Department of Civil Engineering, Michael Okpara University of Agriculture, Umudike, Nigeria; 3 Department of Civil Engineering, Kampala International University, Kampala, Uganda; 4 School of Science and Technology, Hellenic Open University, 26335 Patras, Greece

**Keywords:** Engineering, Materials science

## Abstract

Studying the rheological behavior of concrete, especially self-compacting concrete is vital in the design and structural integrity of concrete structures for design, construction, and structural material sustainability. Both analytical and numerical techniques have been applied in the previous research works to study precisely the behavior of the yield stress and plastic viscosity of the fresh self-compacting concrete with the associated flow properties and these results have not been systematically presented in a critical review, which will allow researchers, designers and filed operators the opportunity to be technically guided in their design and model techniques selection in order to achieve a more sustainable concrete model for sustainable concrete buildings. Also, the reported analytical and numerical techniques have played down on the effect of the shear strain rate behavior and as to reveal the viscosity changes of the Bingham material with respect to the strain rate. In this review paper, a critical study has been conducted to present the available methods from various research contributions and exposed the inability of these contributions to revealing the effect of the shear strain rate on the rheological behavior of the self-compacting concrete. With this, decisions related to the rheology and flow of the self-compacting concrete would have been made with apt and more exact considerations.

## Introduction

**Figure 1:**
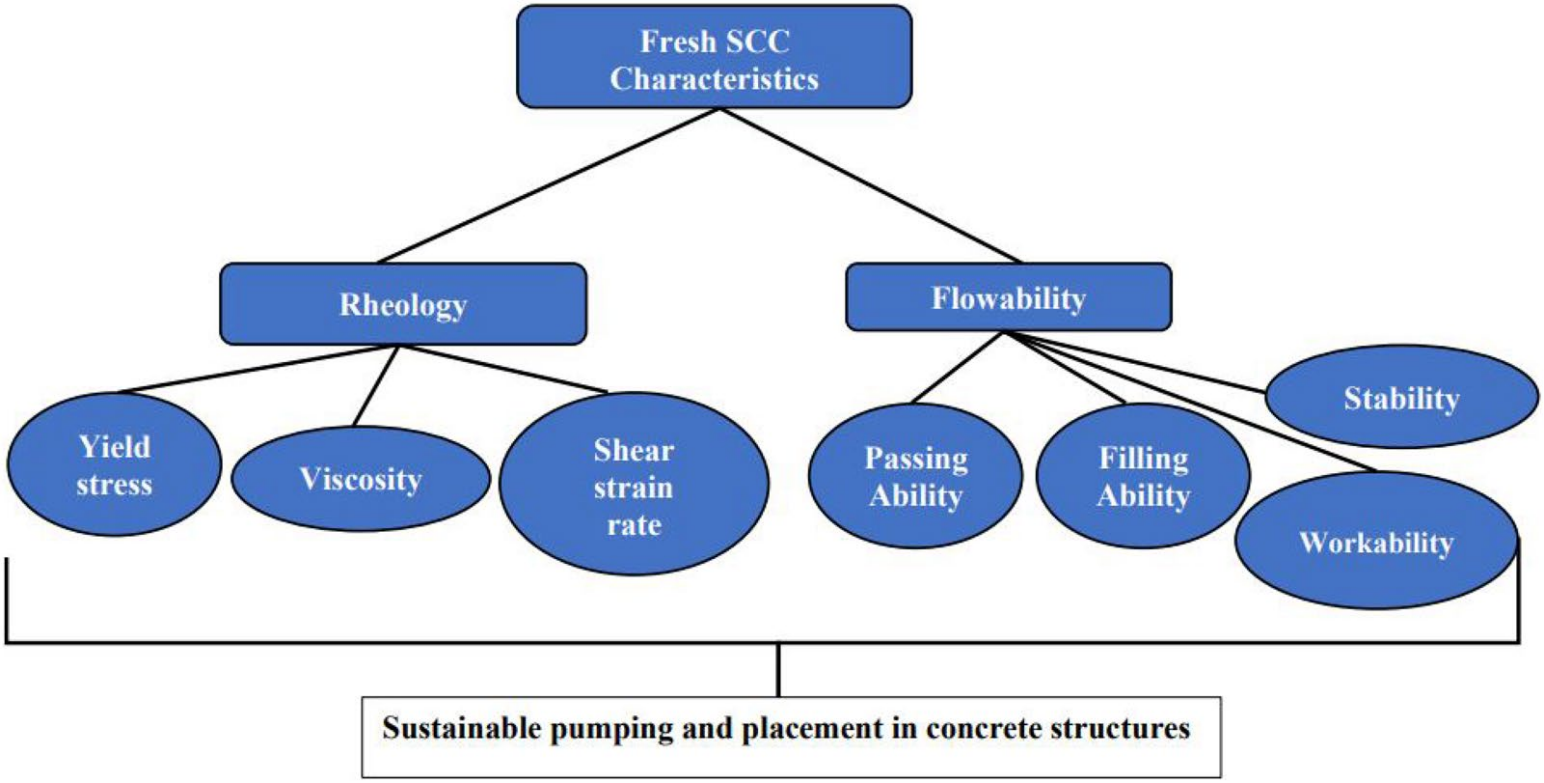
The rheological properties of fresh self-compacting concrete for sustainable handling during concrete structures construction.

**Figure 2:**
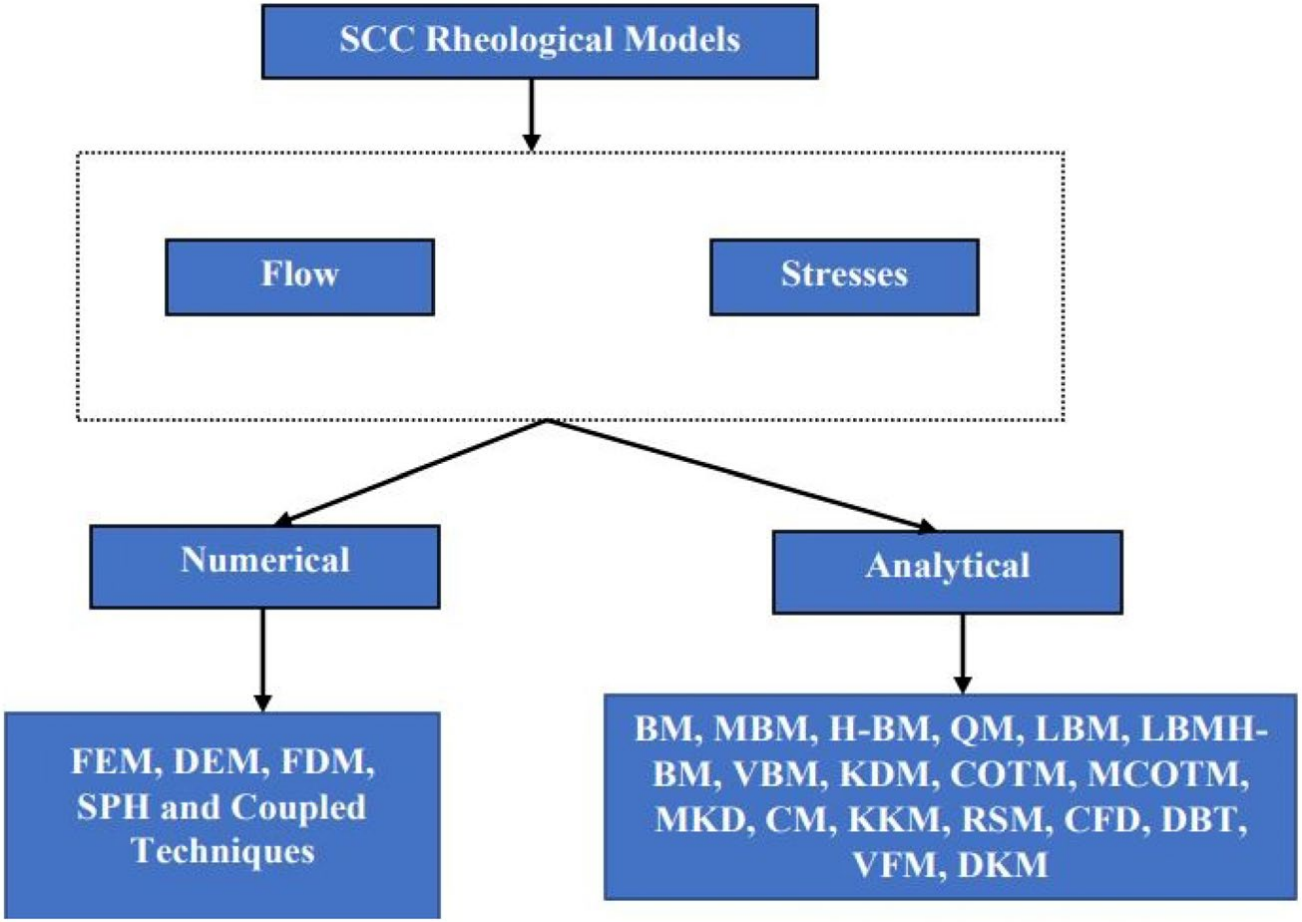
Applied analytical and numerical model techniques for the concrete rheological studies and modeling.

The concept of rheology is an important one in the production and handling of fresh concrete for the sustainability of the building industry^1–3^. This concept includes the study of the stresses (yield stress), viscosity, and flow characteristics which are the physical outcome of the stress inputs^2–5^ as depicted in Fig. 1. This figure shows the two major phases in the characteristic behavior of self-compacting concrete (SCC) as it progresses from the allowable deformation under stresses (shear strain rate, yielding and plastic viscosity) to its fluidic state of flowing (flowability). For the concrete to flow through the structural forms and reinforcement, there are allowable stresses, be it yield stress or viscosity or both and these are of high consideration in the design of the behavior of concrete materials, especially in the case of a self-compacting concrete^6–8^. These properties are studied both in the laboratory using experimental methods and also by the application of mathematical models to represent the field and laboratory conditions under the influence of established boundary conditions^7–9^. In the laboratory/field studies, the V-funnel, L-box, J-ring, Orimet, etc. apparatuses have been developed to study the rheological behavior of the self-compacting concrete (SCC) and this has been done with very wide applications according to the EFNARC requirements^10^. Also, various analytical and numerical techniques have been developed to represent these rheological behaviors of the self-compacting concrete for the purposes of design and construction of sustainable building structures. Most of the analytical and numerical methods in use today have been identified as follows: analytical techniques: Krieger–Dougherty model (KDM), modified Krieger–Dougherty model (MKDM), Chateau–Ovarlez–Trung model (COTM), modified Chateau–Ovarlez–Trung model (MCOTM), Elastic–plastic damage model, Navier–Stokes (NS), Robertson–Stiff Model (RSM), Casson model (CM), De Kee model (DKM), Yahia and Khayat model (YKM), Quemada model (QM), Vom Berg model (VBM), and numerical techniques: Finite Element Method (FEM), Discrete Element Method (DEM), Material Point Method (MPM), Smoothed Particle Hydrodynamics (SPH), etc. as presented in Fig. 2. In the last two decades, these techniques have been used to study and model self-compacting concrete rheological behavior and this has shaped the design and handling of concrete structures in terms of delivery and performance of these structures^9–11^. In this research paper, a critically systematic review has been conducted on the constitutive relations applied in modeling the flow of self-compacting concrete (SCC). This is to further explore the inputs made by various researchers and the applied techniques in the research cause with a view to presenting a synthesized idea of this subject to the research world for ease of application and flexibility in the choice of methods to apply both in their future research efforts and in the field.

## Numerical rheological models in SCC

### Finite element, discrete element and finite difference methods

Dufour and Pijaudier-Cabot^12^ used a homogenous approach integrated into the finite element numerical method to model the flow of the self-compacting concrete considering the Lagrangian integration point (LIP). The material behavior types under time and space motion were observed in this model considering non-linear and time-dependent properties. Also, the interfaces for the material based on the Bingham behavior were monitored. The rheology characteristics of three different concrete materials from experiments were used to compare the results of the LIP-FEM model outcomes. The properties observed and reported in that work were the yield stress, the slump flow spread and the L-box flow of the studied self-compacting concrete. The results show strong agreement between laboratory and model values. It can be observed further that the shear strain rate behavior of the modeled material was not reported as to reveal the viscosity changes of the Bingham material. Hoornahad and Koenders^13^ investigated the grain-paste-grain interaction response in a fresh self-compacting concrete mix slump flow by using the discrete element method (DEM). The explicit description of the interaction of the materials in the mix as a 2-phase paste bridge-system in a mutual interaction influenced by the mix composition with the excess paste theory indulgence was further considered in that research report. The results of the DEM model agreed with the experimental values from the slump flow test. In a following research report, Zhang et al.^14^ investigated the effect of the irregular shaped coarse aggregates on the rheological characteristics of fresh self-compacting concrete. That research paper reported that the shape of aggregates is polytropic as the shapes are not controlled by any known environmental factor except, they are manufactured, hence the particles of a spherical shape are not however engineering in nature and analysis. Further, the slump and L-box flow configurations were adopted in thatstudy and both experimental and simulated situations were used to compare the results. Also, the Bingham material law was observed to study the behavior of the yield stress and the plastic viscosity of the studied models. Finally, the results of the models and the experiments agreed by an insignificant margin. In that paper, a nearly full investigative study was conducted to study the yield stress, viscosity, passing ability, and workability, however, the filling ability and the resistance to segregation of the modeled concrete material were not covered in that report. Again, the DEM has been applied by Mechtcherine et al.^15^ to study the theory and applications in the flow of the self-compacting concrete. The focus of that study^15^ was to show the ability of the DEM interface to model the flow of the concrete. The results showed a strong correlation between model values and standard conditions. In an extended study following the previous work on the theory and application, Mechtcherine et al.^16^ further studied the fresh concrete simulation by using the DEM. The filling ability, passing ability, and workability considering the geometrical conditions of the mixes were observed in that study. The results of the extended DEM model showed strong agreement with the values of the experimental studies. Li et al.^17^ also appliedthe DEM to study the flow of the self-compacting concrete by adopting the slump cone and the J-ring configurations. In that report, a lot of micro-level parameters of the DEM concrete model based on particle–particle and particle-geometry interface were measured, which included the coefficient of restitution, coefficient of rolling friction, coefficient of static friction, and the energy of the surface. High-precision glass spheres were used in place of aggregates to overcome the effect of aggregateshape and sizes on the models. The results of the model showed consistent agreement between the initial stage, rapid stage, and slow stage models of the concrete flow and experimental values. Cui et al.^18^ investigated the influence of coarse aggregate shape on the flowability of the self-compacting concrete by using the constitutive abilities of the DEM. The shape descriptors of needle-shaped coarse aggregates were used to establish the shape of the added material in the model mixes. The flow process through the L-box experimental configuration for the flowing concrete was adopted in this model. The material addition procedure was adopted for continuous incorporation of the aggregate with needle-shapes and the effect on the concrete flow was monitored and recorded. The results of the model showed a consistent correlation with laboratory values and espoused the relevance of aggregate selection during design and infrastructure field operations. Meanwhile, the study reported in that research paper failed to present extensive models based on the flow through other concrete geometries especially the slump and V-funnel so as to study the rheological characteristics of the modeled concrete. In the following research paper by Williams et al.^19^, the descriptors of the aggregate particle shape have been assessed by a digital image segmentation technique and the DEM has been deployed to model the flow effect considering the particle shape parameters. Once more the effect of the aggregate particle shape on the rheological behavior of the self-compacting concrete has been observed and presented in a DEM model outcome, which agrees with experimental records.

### Smoothed particle hydrodynamics

Kulasegaram et al.^20^ studied the modeling of the self-compacting concrete slump flow by making use of the Lagrangian particle-based technique known as the smoothed particle hydrodynamics (SPH). That research was made of with or without the short steel bars technique. The incompressible flow nature of the non-Newtonian concrete materials explained by the Bingham material model was simulated to study the flow of the concrete by using the shear stress to shear strain rate approach and smoothening out the material behavior kink. The aggregates and the steel bars were treated in this model execution as rigid bodies and slender rigid fibers respectively to measure the viscosity of the concrete material through a micromechanical model. The mass conversion incompressibility and the Navier–Stokes were the fundamental equations solved by the SPH interface in this modeling operation. The procedure to securing the solution of this model applied the fractional steps of the prediction-correction technique without pushing the incompressibility state in the runs of the prediction which was satisfied with a divergence free-space. The model results agreed strongly with the allowable slump flow values from literature and standard design requirements and this supported the capability of the SPH to model the rheology and flowability of the self-compacting concrete in the fresh state. However, the yield stress was not studied and reported in that research paper. Deeb et al.^21^ applied the configuration of the L-box in a 3D SPH modeling of the self-compacting concrete flow in a with or without steel fibers’ structure. The simulation emphasis on the without fibers was on the aggregate sizes’ distribution throughout the flow time and domain while the orientation and distribution of the steel fibers were considered the major focus in the with fibers structure. To validate the model, the results were matched with laboratory results from the blocking ratio experiments. The results of the model were consistent with the laboratory values thereby confirming the capability of the SPH to model the passing ratio as the concrete passing ability through reinforced structural members during handling and placement. The model further presented the best orientation for the cut fibers for a more sustainable passing ability of the studied self-compacting concrete. However, the rheology and the filling ability of the fresh concrete were not studied in the research work, which would have presented more comprehensive data for designers and constructors. Deeb et al.^22^ went further to apply the configuration of the Slump cone in a 3D SPH modeling of the self-compacting concrete slump flow in a “with or without” steel fibers’ structure. The simulation emphasis on the “without” fibers was on the aggregates larger than or equal to 8 mm sizes’ distribution throughout the flow time and domain while the orientation and distribution of the steel fibers were considered the major focus in the with fibers structure. To validate the model, the results were compared with laboratory results from the slump flow experiments. The results of the model were consistent with the laboratory values thereby confirming again the capability of the SPH to model the slump flow as the concrete workability and dynamic stability through reinforced structural members throughout the handling and placement. The model further presented the best orientation for the cut fibers for a more sustainable resistance to segregation and workability of the studied self-compacting concrete. However, the rheology based on viscosity and yield and the filling and passing abilities of the fresh concrete were not studied in the research work, which would have further presented more comprehensive data for designers and constructors during field application. Alyhya et al.^23^ applied a 3D SPH technique on the V-funnel configuration in the flow time modeling considering the Bingham-type material model for a non-Newtonian concrete fluid flow. The results of this model agreed favorably with those from the experimental exercise and confirm the capability of the 3D SPH to model the discharge time or the filling ability of self-compacting concrete. Al-Rubaye et al.^24^ also applied the 3D Lagrangian particle-based modeling technique on the L-box configuration to optimize the passing ability of the self-compacting concrete of the Bingham-type behavior, which was coupled with the momentum and the equations of continuity to predict the flow. The focus of the model execution was on the profile of the free surface, flow times, and the “bigger than or equal to 8 mm” aggregates distribution throughout the flow. The experimental results of the actual tests conducted in the laboratory were compared with the model responses in the results were consistent with the allowable values and the model methodology was confirmed suitable to model the passing ability and the aggregate distribution during the concrete handling and placement. Dhaheer et al.^25^ reported the flow behavior modeling in a J-ring concrete configuration using the mesh-free smoothed particle hydrodynamics. The model was simulated from the time the ring was removed to the time the flow stopped. The continuity and the Lagrangian momentum expressions were incorporated in this simulation of the concrete flow through the gaps between the reinforcements also considering aggregate distribution throughout the flow. The results revealed that the simulated passing ability of the mixes conforms to allowable values for the self-compacting concrete and confirms the ability of the SPH methodology to model the passing ability and homogeneity of aggregates during concrete handling. Badry et al.^26^ presented the yield stress and flow behavior through a Slump cone configuration for a self-compacting concrete by using the SPH modeling tool. The simulation was executed for between lifting the cone and stopping of the flow spread. The model work in that research work was focused on observing the ability to forecast the yield stress of the concrete from measured flow times; T-500 and T-stop considering a known plastic viscosity value and also the uniform spread of the 8 mm and larger aggregates at the end of the flow. Results of the model show that the yield stress at the measured times and the aggregate distribution at the end of the flow were consistent with measured values from the laboratory and this confirms also the capability of the SPH to model the slump flow and aggregate distribution throughout the flow situation in self-compacting concrete. Lashkarbolouk et al.^27^ investigated the self-compacting concrete flow simulation through the L-box configuration to study the passing ability of the mix for a non-Newtonian Bingham behavior considering a linear shear to strain rate ratio embodied with the material’s yield stress and plastic viscosity. The results showed values consistent with available literature and laboratory results. It further posited that the allowable viscosity to be considered for self-compacting concrete works lies between 50 and 270 Pa s. These results again confirm the capability of the SPH to model flow and rheologysituations of the fresh self-compacting concrete. Lashkarbolouk et al.^28^ has again applied the V-funnel self-compacting concrete configuration in the modeling of the flow and the rheological behavior of the concrete based on the 2D SPH technique. A homogenous Bingham fluid flow consistency was considered in this time of discharge model exercise. Yield stresses and viscosities were predefined in this model based on the requirements of the EFNARC standard for the self-compacting concrete. The filling ability SPH model through the V-funnel values compared well with the standard values and this result showed the SPH capability to model the flowability of the self-compacting concrete for a sustainable concrete placement and handling operation. However, this was executed based on predetermined rheological values of the fresh concrete flow condition. Further, reinforced concrete structures are mainly the reason for the development of the self-compacting concrete for ease of passing through the blocking effects of the bar under different orientations and that research would have been stronger with the incorporation of the passing ability model through the L-box. Wu et al.^29^ have investigated the capabilities of the enhanced Lagrangian particle-based SPH (eSPH) in the modeling of the flow by using the L-box and slump flow configurations of the self-compacting concrete considering surface deformation, convergence, and fragmentation problems. The results of the 2D eSPH showed great matching results with values from available literature and standard requirements. It further showed satisfactory convergence behavior with the material model execution. However, the viscosity and the filling ability behavior of the studied concrete were not observed or reported in that research paper. Furthermore, Tran-Duc et al.^30^ investigated the effect of coarse aggregate on the flowrate of the self-compacting concrete by using the 2-phase smoothed particle hydrodynamics (SPH) technique considering four different coarse aggregate sizes between 8 and 20 mm with a common density of 2800 kg/m^3^. The coarse aggregates were varied to about 0.3 by vol% fraction of the mix. It was reported in that research paper that coarse aggregate alters the homogeneity and rheology response of the self-compacting concrete in its fresh state. The results reported that the increased coarse aggregate up to 24 wt% increased the yield stress and viscosity and this outcome affects the overall flow behavior of the self-compacting concrete due to its mixes’ resistance to flow due to increased viscosity with increased coarse proportion. The model further showed that upon the addition of bigger coarse aggregates greater than 20 mm, the effective yield stress reduced exponentially confirming the average influence of the bigger averaged contact space in the concrete mixes. This outcome seems to be at variance with the available data. Generally, the summary of the studied numerical techniques yet applied in the self-compacting concrete modeling has been presented in Table 1 for emphasis.

**Table 1:** Summary of the numerical techniques in self-compacting concrete modeling.

Literature	Numerical technique	Rheology	Flowability
Dufour and Pijaudier-Cabot^12^	LIP-FEM	Yield stress	Slump and L-box flows
Hoornahad and Koenders^13^	DEM	–	Slump flow
Zhang et al.^14^	DEM	Yield stress/viscosity	Slump and L-box flows
Mechtcherine et al.^15^	DEM	–	Flows
Mechtcherine et al.^16^	DEM	–	Flows
Li et al.^17^	DEM	–	Slump and J-ring Flow
Cui et al.^18^	DEM	–	L-box flow
Williams et al.^19^	DEM	–	Flows
Kulasegaram et al.^20^	3D SPH	Viscosity	Slump flow spread
Deeb et al.^21^	3D SPH	–	L-box passing ability
Deeb et al. ^22^	3D SPH	Segregation resistance	Slump flow/workability
Alyhya et al. ^23^	3D SPH	–	V-funnel/filling ability
Al-Rubaye et al. ^24^	3D SPH	–	L-box passing ability
Dhaheer et al. ^25^	SPH	–	J-ring flow spread
Badry et al. ^26^	SPH	Yield stress/segregation	Slump flow spread
Lashkarbolouk et al.^27^	SPH	Viscosity	L-box passing ability
Lashkarbolouk et al.^28^	2D SPH	Yield stress/viscosity	V-funnel flow
Wu et al.^29^	2D eSPH	Shear stress/strain	Slump and L-box flow
Tran-Duc et al.^30^	2-ph SPH	Yield stress/viscosity	Flow rate

## Constitutive analytical rheological models in SCC

### Bingham, modified Bingham and Herschel–Bulkley models

Güneyisi et al.^31^ studied the application of the Herschel–Bulkley model (H–BM) and the modified Bingham model (MBM) in the evaluation of the rheological characteristics of a fresh rubberized self-compacting concrete at a fixed water-to-binder ratio of 0.35 and total binder density of 520 kg/m^3^. Meanwhile, 30 wt% of fly ash belonging to class F was included by wt% of the total binder in the mixes used for the model exercise with a rubber percentage of 5–25 wt% as the added component. A rheometer of the ICAR specification was used to monitor the rheological properties related to the workability of the self-compacting concrete mixes. The results showed that the increase in the rubber produced a shear thickening, higher exponent (ή) of the H–BM, and higher coefficients (c/μ) of the MBM. However, the flowability potentials of the self-compacting concrete mixes were not modeled to determine their passing and filling abilities, which is prominent in the working of the flowing concrete structures. In yet another study, Güneyisi et al.^32^ utilized nano silica (NS) and fly ash (FA) as high-range water reducers (HRWR) and viscosity modifiers in the production of the self-compacting concrete and once again further applied the Herschel–Bulkley model (H–BM) and the modified Bingham model (MBM) to model the rheological behavior of the concrete produced at a fixed water-to-binder ratio of 0.33 and density of 570 kg/m^3^. FA was used up to 75 wt% by wt% of the total binder to replace the ordinary cement in this exercise. The experimental configurations utilized in the protocol were the slump flow spread, slump flow time at 50 mm, V-funnel flow time, and the L-box blocking ratio. These apparatuses measured the workability, yield stress, passing and filling abilities, and segregation resistance. The shear rate from this model was observed with the increase in the values of the exponents and the coefficients of the H–BM and MBM respectively. The models and the experimental values were compared afterwards and the results agreed within an acceptable margin as the increase in the FA and NS improved the rheology of the studied concrete in line with design requirements. Feys et al.^33^ applied a time-independent approach to model the fresh behavior of the self-compacting concrete by using the Herschel–Bulkley model (H–BM) and the Bingham model (BM), modified Bingham model (MBM). Whereas the BM produced negative yield stresses and the H–BM suffered mathematical restrictions in terms of the models’ execution, the MBM showed superior performance in modeling the rheological properties of the studied concrete with appropriate indices to define the concrete model shear thickening in terms of the shear rate coefficients (c/μ). In that work, the MBM has proven to be more accurate and appropriate in handling the rheology and flow of the self-compacting concrete considering the conditions and assumptions of the present research work.

### Lattice Boltzmann method (LBM)

Li et al.^34^ investigated the application of the LBM in the simulation of the slump flow of self-compacting concrete using the slump cone configuration for five different groups of fresh concrete mixes. The rheological characteristics in that research work were optimized with the Herschel–Bulkley (H–B) and the Bingham material models. The fitting approaches of the linear and nonlinear derivation of equations and the rheometer measurements were applied in the determination of the microparameters of the fresh concrete and their optimization. The H–B model showed its superior accuracy in the rheological behavior estimation and optimization of the concrete. The results further revealed a higher degree of errors in the Bingham model than in the Herschel–Bulkley (H–B) model. Also, the influence of the rheological response on the concrete flow time, velocity, shear strain rate, flow rate, and concrete placement contour appearance was revealed in the models. However, the study did not observe the viscosity changes as a non-Newtonian fluid model of the Bingham type at different rates of shear strain. This further would have given a clearer understanding of the micro-behavior of the strain stresses during the flow model of the self-compacting concrete under gravitational flow and plastic deformation along the flow path. A multiple relaxation time optimized LBM has been applied by Qui and Han^35^ in the flow of self-compacting concrete based on the 3D format and the model of discrete velocity and a constitutive mode of the H–B was adopted in the model execution as the modifier. The slump flow and the L-box passing configurations were used in the study and the modeled numerical values were matched with the values from the available literature. The model proved its capability to forecast the flow behavior of the self-compacting concrete as it compared well with the measured experimental values from the literature. Once more the rheological characteristics of the studied concrete of this model operation have been avoided in the report. Mu et al.^36^ investigated the flow of the self-compacting concrete through a U-box configuration by using the LBM combined with the H–B model framework. The yield stress, power law index, and consistency index were studied with the combined LBM–H–B model framework. The simulation results though with a 7% average error showed high accuracy. The LBM–H–B model further reported that the yield stress was the determinant factor towards the efficient flow and passing ability of the studied self-compacting concrete and that its increase resulted in the weaker passing ability in the concrete flow regime. The results also reported that the indices of the power law and the consistency did not present any significant effect on the studied concrete passing ability and that the total increase in the values of yield stress, power law index, and consistency index reduced the shear velocity in a shear-thickening consistency. Meanwhile, Kabagire et al.^37^ adequately applied the Krieger–Dougherty (KD) and Chateau–Ovarlez–Trung (COT) models in evaluating the plastic viscosity and the static yield stress of the self-compacting concrete mixes at water-to-powder (w/p) ratios of 0.30 and 0.35. Also, the rheological characteristics’ responses to the incorporation of the solid fractions and particle configurations were observed during the experimental phase. The results showed the adequacy of the KD and the COT models in predicting the concrete rheology for effective placement during the pumping and concreting of concrete structures. In an attempt to present more efficient model versions of the Krieger–Dougherty (KD) and Chateau–Ovarlez–Trung (COT) models, Kabagire et al.^38^ presented the modified Krieger–Dougherty (MKD) and modified Chateau–Ovarlez–Trung (MCOT) models in the predictions of the flow and rheological characteristics of the self-compacting concrete considering the incorporation of manufactured sand, volume of paste (VP), volume of sand (VS), paste-to-sand ratio (VP:VS), w/b ratio and the content and type of coarse aggregates. The results showed that the MKD and the MCOT showed the most satisfying outcome in the prediction of the rheology of the fresh concrete, which further reported that the VP:VS and the w/b influence the model’s performance more than the other parameters incorporated into the exercise. Huang et al.^39^ investigated the shear thickening behavior of the self-compacting concrete made from high volume superplasticizer (SP) and air-entraining agent (AEA) by using the modified Bingham model (MBM). The results showed that at intensified shear thickening of the modeled concrete, the yield stress and the plastic viscosity significantly decreased, which resulted in the increase in the wt% of the SP while at increased AEA, the YS increased with a decrease in PV whereas high air content weakened the shear thickening, which reduced to zero leading to shear thinning behavior of the model at the air proportion reached up to 8.7%. This model showed that air-entraining is an effective means of improving the performance of the rheological characteristics of the self-compacting concrete and of course a sustaining concrete placement. Meanwhile, the passing and filling abilities of the concrete were not modeled in the MBM protocol. In a more comprehensive model exercise to study the behavior of the shear rate of the self-compacting concrete, Campos and Maciel^40^ applied the Bingham model (BM), modified Bingham model (MBM), and the Herschel–Bulkley model (H–BM) considering water-to-cement mass ratios of 0.40 and 0.65 under the influence of the shear rates of 50/s and 100/s and single- and multiple-staged distinct shearing techniques. Further on, experimental data were used to fit the model values. The results showed that models and test methodology exert effects on the evaluation of the rheological parameters of the self-compacting concrete. Meanwhile, the H–BM showed superior performance in the prediction of the rheology of the studied concrete. It further reports that more reliable and applicable results were achieved with the multiple-staged distinct shearing method at the shear rate of 100/s. Rehman et al.^41^ equally conducted another comprehensive model exercise using the Bingham model (BM), modified Bingham model (MBM) and the Herschel–Bulkley model (H–BM), and the Casson model (CM) to predict the yield stress and the plastic viscosity of the self-compacting concrete under different shear rates of 300, 200 and 100/s. Graphene was incorporated in the wt% of 0.03, 0.05 and 0.10 wt% of cement in the concrete mixes. Also, the flow curves trend was observed under the influence of SP. While graphene increase increased the YS and the PV, the effect of the SP was different. The YS increased and the PV decreased with an increase in the shear rates on the concrete model. Finally, the H–BM, which produced the lowest computation error outclassed the BM, MBM, and CM thereby proving to be the best framework to predict the rheological behavior and flow trend of the self-compacting concrete mixed with graphene and SP and subjected to shear rates between 300 and 100/s. Once more the passing and filling ability of the model concrete mixes were not studied in this exercise leaving much to be done to evaluate the flowability potential of the concrete. In an effort to pursue the net-zero protocol of the UNSDGs and COP27 for the year 2050 towards carbon neutrality in engineering design and infrastructural development, Taleb et al.^42^ investigated the effect of natural pozzolana (NP) as a partial replacement for Portland cement in self-compacting concrete mixes. This procedure was modeled with MBM and H–BM techniques considering a slump test configuration to study the YS and PV of the mixes. Both models (MBM and H–BM) showed a satisfactory shear thinning behavior of the mixes with natural pozzolana (NP) addition to the mix. But in terms of the rheological properties, the MBM seemed to perform better this time. It further reported that at 30 wt% replacement of cement with NP, the YS and PV values showed acceptable ranges required by standard design conditions and further reduced carbon emission by 40%. Meanwhile, the flowability characters were not considered in the model execution leaving yet another vacuum for further research studies to evaluate the flowability indices of the natural pozzolana-based self-compacting concrete. The YS and PV have been evaluated in another work by Petersson et al.^43^ by using the basic Bingham model considering different fillers, viscosity enhancers, and superplasticizers. The slump flow and the L-box configurations were adopted in that work to model the rheological behavior and the passing ability and workability. With the strong agreement between the model values and the experimental values, the model has shown to be appropriate in predicting the rheological behavior of the self-compacting concrete with different fillers, VMA, HRWR, etc. incorporated. Furthermore, Zhaidarbek et al.^44^ conducted a complex model transition from the Khatib-Khayat model (KKM) to the H–BM and MBM in the evaluation of the flow rate to pressure drop relationship in self-compacting concrete pumping considering the oiling layer and the shear thickening characteristics of the studied fresh concrete. In this process, a new method was developed to determine the flow rate-to-pressure drop relationship based on the Hagen–Poiseuille flow incompressible concrete fluid state conditions. The shear rate and velocity distributions based on the H–BM and MBM were observed to graphically produce the volumetric flow rate-loss of pressure behaviors. The results showed an innovative approach of hybridization of the Khatib–Khayat model (KKM) into the H–BM and MBM for a more appropriate and efficient prediction of the flow rate of the self-compacting concrete during design and concrete placement. However, the filling and passing abilities as well as the yield stress and viscosity of the studied concrete were not put into consideration in that research report. Also, the response of the concrete behavior with respect to the addition of different fillers, VMA, HRWR, etc. was not considered. Further on, an entirely new approach known as the volume of fluid model (VFM) was used by Shin et al.^45^ to predict the flow behavior and the yield stress of the self-compacting concrete based on the single-fluid simulation, which accommodated the partial instability or consolidation of the coarse aggregates in the modeled mixes. The L-box configuration was adopted in this model for passing ability flow simulation. In the end, the results were used to update the rheograph, which was used to study the rheological characteristics of the self-compacting concrete for the purposes of efficient design and sustainable construction. Hosseinpoor et al.^46^ sufficiently applied the computational fluid dynamics (CFD) based on the Dam Break Theory (DBT) in a seemingly different analytical approach to model and simulate the flow profile for a self-compacting concrete using a modified L-box configuration set-up. The influence of gravity on the flow conditions of this flowing concrete model set-up into the horizontal section was observed by filing the vertical section of the modified L-box set-up at 50 and 110 cm heights with concrete mixes of low-to-high segregation resistance levels. Also, three stop times of 1, 5, and 15 min were used to evaluate the effect of the static segregation on the flow conditions of the modeled self-compacting concrete. The studied concrete was assumed to have linear and nonlinear flow characteristics, which were described by adopting the rheological models of Bingham (linear) and Herschel–Bulkley (nonlinear) considering the relationship coefficients and exponents, respectively. The results of the analytical models based on the DBT showed accurate predictions which agreed with the values of the modified L-box experimental results. It was further reported that the Herschel–Bulkley (nonlinear) model led to better analytical predictions than the linear Bingham model. However, the yield stress and the shear rate conditions were not determined in that work. Furthermore, on the analytical models’ application in the prediction of the flowability characteristics of the self-compacting concrete, Onyelowe et al.^47^ and^48^, respectively applied the L-box and V-funnel concrete experimental set-up configurations in the forecast of the blocking ratio and the flow time of the studied concrete. These models were responsible for the modeling of the passing ability and the filling ability respectively of the studied concrete by making use of Bernoulli’s and the flow continuity theories (BT and CT). The geometry of the two configurations was basic in these analytical models. For the L-box blocking ratio model reported by Onyelowe et al.^47^, embedded steel bars were considered under shear stress and pressure conditions of the self-compacting concrete. Sixty seconds (60 s) elapse-time was used before opening the shutter between the vertical and horizontal compartments of the L-box and the sum of the forces between the upstream and downstream was evaluated under the continuity theory (CT). The vertical stress with respect to the friction stress between the concrete particles and the walls was determined and incorporated into the flow mechanism of the analytical model. The yield stress distribution on the flow path was graphically presented and the passing which fell within an acceptable range according to the standard requirements provided by EFNARC was obtained. With this flow output, the L-box model according to the BT and CT proved to be appropriate in the prediction of the passing ability of the self-compacting concrete for industry use and application in infrastructure designs. For the V-funnel flow time (filling ability) model reported by Onyelowe et al.^48^, The BT and CT were applied on the V-funnel with an outlet duct of 75 × 65 mm and effective dimension of 515 × 600 mm according to EFNARC standard. The principle of flow convergence was merged in the V-funnel flow model continuity behavior. The time taken for the studied self-compacting concrete to flow through an elemental height dh of the funnel configuration was considered for the weight of the concrete, the downward acceleration due to gravity, and the upward friction force which accounted for the dynamic viscosity of the mix. The model results showed acceptable flow time limits between 5 and 25 s agreeable with the requirements of the EFNARC. Again, the BT and CT showed their coupled capability to predict the filling ability conditions of the self-compacting concrete through the V-funnel set-up. A similar method of model called the new rheological model cell method (NRM-CM) has been applied by Mahmoodzadeh and Chidiac^49^ to predict the yield stress and the plastic viscosity of self-compacting concrete. Benaicha et al.^50^, developed a v-funnel innovative device applied to quantify the rheological behaviors of the self-compacting concrete (SCC), which are basically yield stress and plastic viscosity, which determines the micro-behavior of the SCC flow. Tests were conducted to model/caliberate the system using the provisions of EFNARC and the recommendations of AFGC. Comparative results from this study show that the SCC characterization can successfully be carried out using the method from this research case and its empirical suggestions. Benaicha^51^ also applied empirical correlation method based on abacus to develop concrete flow characterization techniques for the self-compacting concrete plastic viscosity and yield stress independent of field operators influences on site. In this case, the v-funnel, l-box and slump cone models were successfully correlated with empirical results based on EFNARC guidlines. Table 2 has presented a summary of the available analytical techniques to date that have been applied in the modeling and characterization of the rheology; shear rate, yield stress, plastic viscosity, etc. of the SCC. Various other admixture and replacement materials have been applied in the production of the self-compacting concrete for the purposes of sustainability especially the discarded sandstone slurry. Metakaolin, rice husk ash, etc.^52–57^. However the research study investigated the workability, strength, water absorption, freeze–thaw resistance and permeable voids of the concrete and did not delve deep into the concrete rheological behavior. Finally, the summary of the constitutive analytical modeling of the rheological states of the self-compacting concrete is presented in Table 2.

**Table 2:** Summary of the analytical techniques in self-compacting concrete modeling.

Literature	Numerical technique	Rheology	Flowability
Güneyisi et al.^31^	H–BM & MBM	Yield stress and shear rate	Slump flow
Güneyisi et al.^32^	H–BM & MBM	Shear rate	Slump, V-funnel, and L-box flows
Feys et al.^33^	BM, H–BM & MBM	Shear rate	Slump flow
Li et al.^34^	LBM	–	Slump flow
Qui and Han^35^	LBM & H–B	–	Slump and L-box flow
Mu et al.^36^	LBM & H–B	Yield stress, power law index, and consistency index	U-box passing ability
Kabagire et al.^37^	KD & COT	Plastic viscosity and yield stress	Slump flow
Kabagire et al.^38^	MKD & MCOT	Plastic viscosity and yield stress	Slump flow
Huang et al.^39^	MBM	Yield stress, viscosity, and shear rate	–
Campos and Maciel^40^	BM, MBM, H–BM	Shear rate	Slump flow
Rehman et al.^41^	BM, MBM, H–BM , & CM	Yield stress, viscosity and shear rate	Flow trend
Talebet al.^42^	MBM & H–BM	Yield stress and plastic viscosity	–
Petersson et al.^43^	BM	Yield stress and plastic viscosity	Slump and L-box flow
Zhaidarbek et al.^44^	KKM to H–BM & MBM	Shear rate	Flow rate
Shin et al.^45^	VFM	Yield stress	Slump flow
Hosseinpoor et al.^46^	CFD-DBT	–	L-box flow
Onyelowe et al.^47^	BT & CT	–	L-box flow
Onyelowe et al.^48^	BT & CT	–	V-funnel flow
Mahmoodzadeh and Chidiac^49^	NRM-CM	Yield stress and plastic viscosity	–
Benaicha et al.^50^	Analytical	Yield stress and plastic viscosity	V-funnel/horizontal channel system
Benaicha^51^	Abacus	Yield stress and plastic viscosity	V-funnel, L-box, and Slump cone

## Conclusions

A critical review of rheological models in self-compacting concrete for sustainable structures has been conducted, which has extensively presented the application of the analytical and numerical techniques previously applied in modeling the yield stress, plastic viscosity, and flow characteristics of the studied concrete. Several techniques were presented with superior outputs. In this research also, the effect of the aggregate proportioning, particle friction, viscosity modifying agent (VMA), superplasticizers, and water-cement ratio on the concrete rheology and flow was also explored.Furthermore, the flows through the slump cone, L-box, V-funnel, Orimet, J-ring, and U-tube apparatuses with respect to the yield stress and viscosity of the fresh self-compacting concrete were reviewed in connection to numerical and analytical models. The following are the conclusions from the critical review of the rheological models.

- The slump cone, V-funnel, and L-box have been prominently used in the estimation of the self-compacting concrete rheological behavior over the other apparatuses due to their relationship with flow shearing.
- The coarse aggregates were found to increase the yield stress and viscosity when used with sizes greater than 20 mm, which reduces the flow efficiency of the fresh concrete.
- The analytical modeling techniques were found to exhibit limitations in the computation of yield stress, viscosity, and the flow properties of concrete due to the inability to handle complex concrete state conditions.
- However, the numerical modeling techniques perform optimally in coupled mathematical computations like the LBM–H–B and SPH model framework for the fresh concrete (SCC) rheology, which showed the highest accuracy so far.
- It is recommended that Orimet flow model be studied by applying novel numerical and analytical methods as a supplimmentary method to the V-funnel, L-box and the slump cone techniques.

### Scope for future research

Future research works in this area should be directed at studying the application different hybrids of the smoothed particle hydrodynamics, which has presented a potential to adapt other techniques in its computational framework in solving this concrete structural problem.

## Data Availability

Data are available within the article.
